# A blood test-derived skeletal muscle index combined with handgrip strength identifies a sarcopenia-related high-risk phenotype in non–dialysis-dependent chronic kidney disease

**DOI:** 10.1093/ckj/sfag336

**Published:** 2026-09-25

**Authors:** Shunsuke Yamada, Shigeru Tanaka, Kenji Harada, Hisako Yoshida, Hiromasa Kitamura, Hokuto Arase, Masumi Shojima, Kazuhiko Tsuruya, Toshiaki Nakano, Tetsuro Ago

**Affiliations:** Department of Medicine and Clinical Science, Graduate School of Medical Sciences, Kyushu University, Fukuoka, Japan; Division of Kidney Care Unit, Kyushu University Hospital, Fukuoka, Japan; Department of Medicine and Clinical Science, Graduate School of Medical Sciences, Kyushu University, Fukuoka, Japan; Department of Nephrology, Kokura Memorial Hospital, Kitakyushu, Japan; Department of Medical Statistics, Osaka Metropolitan University Graduate School of Medicine, Osaka, Japan; Department of Medicine and Clinical Science, Graduate School of Medical Sciences, Kyushu University, Fukuoka, Japan; Division of Nephrology, National Hospital Organization Fukuoka-Higashi Medical Center, Koga, Japan; Department of Medicine and Clinical Science, Graduate School of Medical Sciences, Kyushu University, Fukuoka, Japan; Department of Nephrology, Nara Medical University, Kashihara, Japan; Department of Medicine and Clinical Science, Graduate School of Medical Sciences, Kyushu University, Fukuoka, Japan; Division of Kidney Care Unit, Kyushu University Hospital, Fukuoka, Japan; Department of Medicine and Clinical Science, Graduate School of Medical Sciences, Kyushu University, Fukuoka, Japan; Center for Cohort Studies, Graduate School of Medical Sciences, Kyushu University, Fukuoka, Japan

**Keywords:** chronic kidney disease, handgrip strength, sarcopenia-related phenotype, serum creatinine-cystatin C ratio, skeletal muscle index

## Abstract

**Background:**

Sarcopenia is associated with adverse outcomes in CKD, but direct assessment of skeletal muscle mass is not always available. We examined whether serum creatinine- and cystatin C-derived estimated skeletal muscle index (SMI), combined with handgrip strength, identifies a sarcopenia-related high-risk phenotype in non–dialysis-dependent CKD.

**Methods:**

We derived a new equation for appendicular skeletal muscle mass using bioelectrical impedance analysis as the reference in 100 patients with non–dialysis-dependent CKD. In an independent multicenter cohort of 1403 patients, SMI was estimated using this equation and a published creatinine-cystatin C-based formula. The sarcopenia-related high-risk phenotype was defined as low handgrip strength plus low estimated SMI using sex-specific cutoffs. Outcomes were all-cause, cardiovascular, and noncardiovascular mortality.

**Results:**

During 5 years, 173 patients died, including 43 from cardiovascular causes. Low estimated SMI was identified in 218 patients (15.5%) with the published formula and 312 (22.2%) with the new equation; the corresponding phenotype prevalences were 11.9% and 11.7%. The phenotype was independently associated with all-cause mortality using either formula: adjusted hazard ratios were 1.54 [95% confidence interval (CI), 1.07–2.21] for the published formula and 2.14 (95% CI, 1.52–3.00) for the new equation. Four-category and sensitivity analyses yielded consistent findings.

**Conclusions:**

Combining handgrip strength with creatinine- and cystatin C-derived estimated SMI identifies a sarcopenia-related high-risk phenotype and stratifies mortality risk in non–dialysis-dependent CKD. This approach may support screening when direct muscle mass assessment is unavailable but requires further validation before use as a diagnostic substitute.

## Graphical Abstract

**Graphical Abstract:**
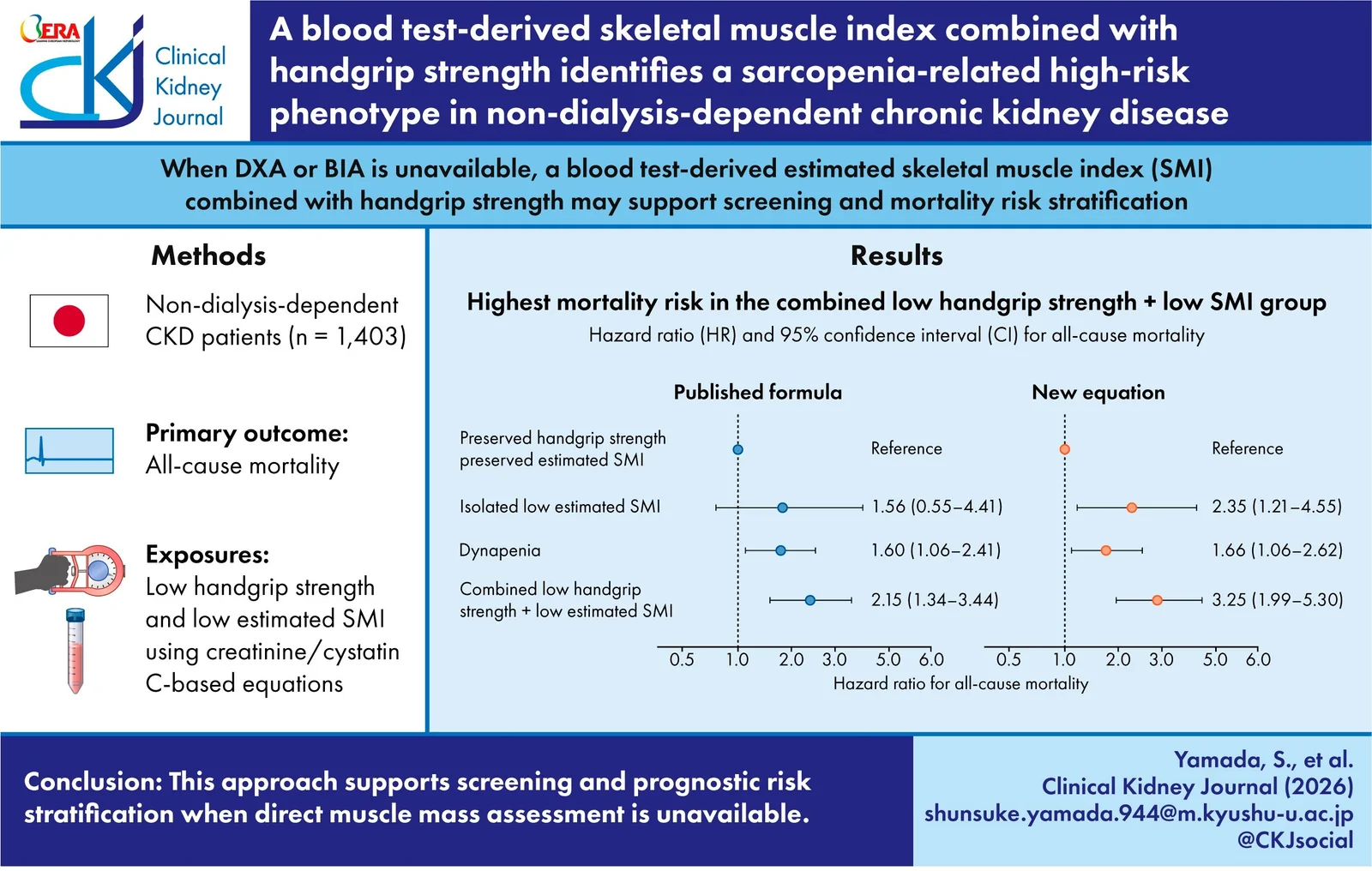

KEY LEARNING POINTS **What was known:**

1. Sarcopenia and muscle-related vulnerability are common in chronic kidney disease and are associated with mortality and other adverse outcomes.
2. Assessment of skeletal muscle mass generally requires dual-energy X-ray absorptiometry, bioelectrical impedance analysis, or computed tomography, which may not be available in routine nephrology practice.
3. Serum creatinine- and cystatin C-based estimates of muscle mass have been proposed, but their value when combined with handgrip strength for risk stratification remains uncertain.

**This study adds:**

1. We developed a new serum creatinine- and cystatin C-based equation to estimate appendicular skeletal muscle mass in non–dialysis-dependent CKD using bioelectrical impedance analysis as the reference.
2. When combined with handgrip strength, blood test-derived estimated SMI identified a sarcopenia-related high-risk phenotype in this population.
3. This phenotype was independently associated with all-cause and cardiovascular mortality and showed modest incremental prognostic value for all-cause mortality beyond conventional clinical markers.

**Potential impact:**

1. This approach may help screen for muscle-related vulnerability when direct muscle mass assessment is unavailable.
2. Because it relies on routine blood tests plus handgrip strength, it could broaden access to prognostic muscle assessment in everyday CKD care.
3. Patients identified by this approach may warrant closer evaluation of nutritional status, muscle strength, physical function, physical activity, and rehabilitation needs, although intervention studies are needed.

## INTRODUCTION

Patients with chronic kidney disease (CKD) have a substantial burden of sarcopenia and muscle-related vulnerability driven by the uremic milieu, chronic inflammation, undernutrition, and reduced physical activity [1, 2]. Sarcopenia is associated with adverse outcomes in CKD, including mortality and functional decline [3–5]. These observations underscore the need for simple, repeatable approaches to assess muscle-related risk in routine CKD care.

The Asian Working Group for Sarcopenia 2019 (AWGS 2019) defines sarcopenia as low muscle mass accompanied by low muscle strength and/or low physical performance [6]. The AWGS 2025 update further emphasized muscle health and age-specific assessment in Asian populations [7]. The European Working Group on Sarcopenia in Older People 2 defines sarcopenia using low muscle strength and low muscle quantity or quality, with consideration of physical performance [8]. Handgrip strength is generally assessed using handgrip dynamometry. Skeletal muscle mass is typically quantified as skeletal muscle index (SMI) using dual-energy X-ray absorptiometry or bioelectrical impedance analysis [6, 8–10]. Because these measurements are not uniformly available across medical facilities, guideline-concordant assessment is often constrained, prompting interest in pragmatic alternatives.

A growing body of evidence indicates that indices derived from serum creatinine and serum cystatin C, particularly their ratio, reflect skeletal muscle mass and function [11, 12]. Prior studies in CKD and other clinical cohorts show that these indices correlate with bioelectrical impedance analysis-derived measures and predict clinically relevant outcomes [13–23]. However, whether blood test-derived estimated SMI, when combined with handgrip strength, identifies a clinically meaningful sarcopenia-related high-risk phenotype in non–dialysis-dependent CKD remains uncertain.

This study had two aims: first, to test whether a blood test-based approach using serum creatinine and serum cystatin C can estimate SMI in patients with CKD; and second, to determine whether low estimated SMI combined with low handgrip strength is associated with mortality. To address these aims, we evaluated the equation proposed by Sunayama *et al*. [17] and developed a new serum creatinine-serum cystatin C-based formula to estimate appendicular skeletal muscle mass in non–dialysis-dependent CKD. We then examined mortality risk under each definition in a prospective multicenter non–dialysis-dependent CKD cohort [24].

## MATERIALS AND METHODS

### Derivation of a new equation predicting appendicular skeletal muscle mass

To develop a new equation that predicts appendicular skeletal muscle mass, we recruited 100 patients with non–dialysis-dependent CKD who were hospitalized for CKD education and assessment of CKD-related complications, independent of the Fukuoka Kidney disease Registry (FKR) study [25]. Data included age, sex, height, body weight, serum creatinine, serum cystatin C, and appendicular skeletal muscle mass measured by bioelectrical impedance analysis using the InBody device (InBody Japan, Tokyo, Japan). A multivariable linear regression model was used to derive a formula. We refer to this equation as the new formula or ‘Yamada’s formula,’ which was used to calculate appendicular skeletal muscle mass based on age, sex, body weight, serum creatinine, and serum cystatin C; then, estimated SMI was obtained by dividing appendicular skeletal muscle mass (kg) by squared height (m^2^).

### The FKR Study design and subjects

The FKR Study is a prospective multicenter cohort study of non–dialysis-dependent CKD patients in Japan. The details have been described elsewhere [24]. In brief, the study population consisted of 4476 outpatients aged 16 years or older who were treated by nephrologists at 12 facilities in Fukuoka Prefecture. All patients were enrolled between January 2013 and March 2017 and were scheduled to be followed for at least 5 years. Among 1505 patients with handgrip strength data, 1403 without missing data were included. The Kyushu University Hospital Institutional Review Board for Clinical Research (No. 469-04) approved the study protocol.

### Primary outcome and exposures

The primary endpoint was all-cause mortality. Cardiovascular and noncardiovascular mortality were secondary endpoints. Low handgrip strength was defined using AWGS 2019 cutoffs of <28.0 kg for men and <18.0 kg for women [6]. Low estimated SMI was defined using AWGS 2019 muscle mass cutoffs of <7.0 kg/m^2^ for men and <5.7 kg/m^2^ for women. Estimated SMI was calculated using Sunayama’s equation and our new equation. The coexistence of low handgrip strength and low estimated SMI was defined as a sarcopenia-related high-risk phenotype. Patients were also divided into four groups according to handgrip strength and estimated SMI: preserved handgrip strength and estimated SMI, isolated low estimated SMI, dynapenia, and combined low handgrip strength and low estimated SMI. Dynapenia was defined as low handgrip strength with preserved estimated SMI. Because direct muscle mass measurement and physical performance were unavailable in the FKR cohort, this operational definition was used for risk stratification and was not considered a complete guideline-based diagnosis of sarcopenia. Sunayama’s formula is as follows [17]: SMI for men (kg/m^2^) = 4.17–0.012 × [Age] + 1.24 × [serum creatinine/serum cystatin C]–0.0513 × [Hemoglobin] + 0.0598 × [Body weight]; SMI for women (kg/m^2^) = 3.55–0.00765 × [Age] + 0.852 × [serum creatinine/serum cystatin C]–0.0627 × [Hemoglobin] + 0.0614 × [Body weight]. In our previous study, the validity of using Sunayama’s formula for non–dialysis-dependent CKD patients as a surrogate for SMI was confirmed [16].

### Covariates and biochemical determinations

The following baseline characteristics were obtained from the medical records: age, sex, presence of diabetes mellitus, history of cardiovascular disease, systolic blood pressure, estimated glomerular filtration rate (eGFR), urinary protein-to-creatinine ratio, blood hemoglobin level, and serum levels of albumin, high-sensitivity C-reactive protein (hsCRP), calcium, and phosphorus. Serum albumin, creatinine, hsCRP, and urinary creatinine were determined at a central laboratory; other biochemical parameters were measured using standard auto-analyzers. Serum creatinine-based eGFR and serum cystatin C-based eGFR were calculated using Japanese equations [26, 27], and their arithmetic mean was used in the current analyses. Handgrip strength was measured using a standard handgrip dynamometer. The mean value of measurements from both hands was used; however, if data from only one hand were available, that value was adopted.

### Statistical analysis

Continuous variables are described as medians with interquartile range and categorical variables as numbers with percentage. Continuous variables were compared using the Mann–Whitney *U* test, and categorical variables using the chi-square test or Fisher’s exact test, as appropriate. *P*-values for descriptive comparisons were presented without formal correction for multiple comparisons. Unadjusted, age- and sex-adjusted, and multivariable-adjusted hazard ratios (HRs) and 95% confidence intervals (CIs) for mortality were estimated using Cox proportional hazards regression analysis. The multivariable-adjusted models included age, sex, presence of diabetic nephropathy, history of cardiovascular disease, systolic blood pressure, eGFR, log-transformed urinary protein-to-creatinine ratio, blood hemoglobin, serum albumin, and log-transformed hsCRP. Proportional hazards were assessed using Schoenfeld residuals and log(-log) plots. Restricted cubic splines with four knots were used to assess nonlinear associations between handgrip strength, estimated SMI, and mortality.

Subgroup analysis stratified by baseline clinical characteristics was also conducted. As secondary analyses, the impact of low handgrip strength and low estimated SMI on mortality was separately determined. Incremental prognostic value for all-cause mortality was assessed by comparing a base clinical model with an extended model additionally including the phenotype, using the C-index, Akaike information criterion, and likelihood-ratio test. The base model included conventional prognostic factors used in the multivariable Cox model. These analyses were intended to quantify added prognostic information, not to establish diagnostic accuracy. Two post-hoc sensitivity analyses were performed: applying AWGS 2025 age-specific cutoffs among participants aged 50 years or older [7], and excluding CKD stage G5. A two-tailed *P*-value <.05 was considered statistically significant. Statistical analyses were performed using R software version 4.2.2.

## RESULTS

### Development of a new equation estimating appendicular skeletal muscle mass

Mean patient age in the derivation cohort was 67 years. Sixty patients were male and 24 had diabetic nephropathy. Median serum creatinine and serum cystatin C levels were 2.82 mg/dl and 2.73 mg/l, respectively. By applying multivariable linear regression, we developed the following new formula: appendicular skeletal muscle mass (kg) = 4.21–0.027 × [Age] + 3.32 × [Sex (0 = female, 1 = male)] + 0.21 × [Body weight] + 1.23 × [serum creatinine/serum cystatin C]. In this equation, age is expressed in years, body weight in kg, serum creatinine in mg/dl, and serum cystatin C in mg/l. The correlation coefficient between bioelectrical impedance analysis-measured and estimated appendicular skeletal muscle mass was 0.818, and the coefficient of determination was 0.67 (Fig. 1).

**Figure 1:**
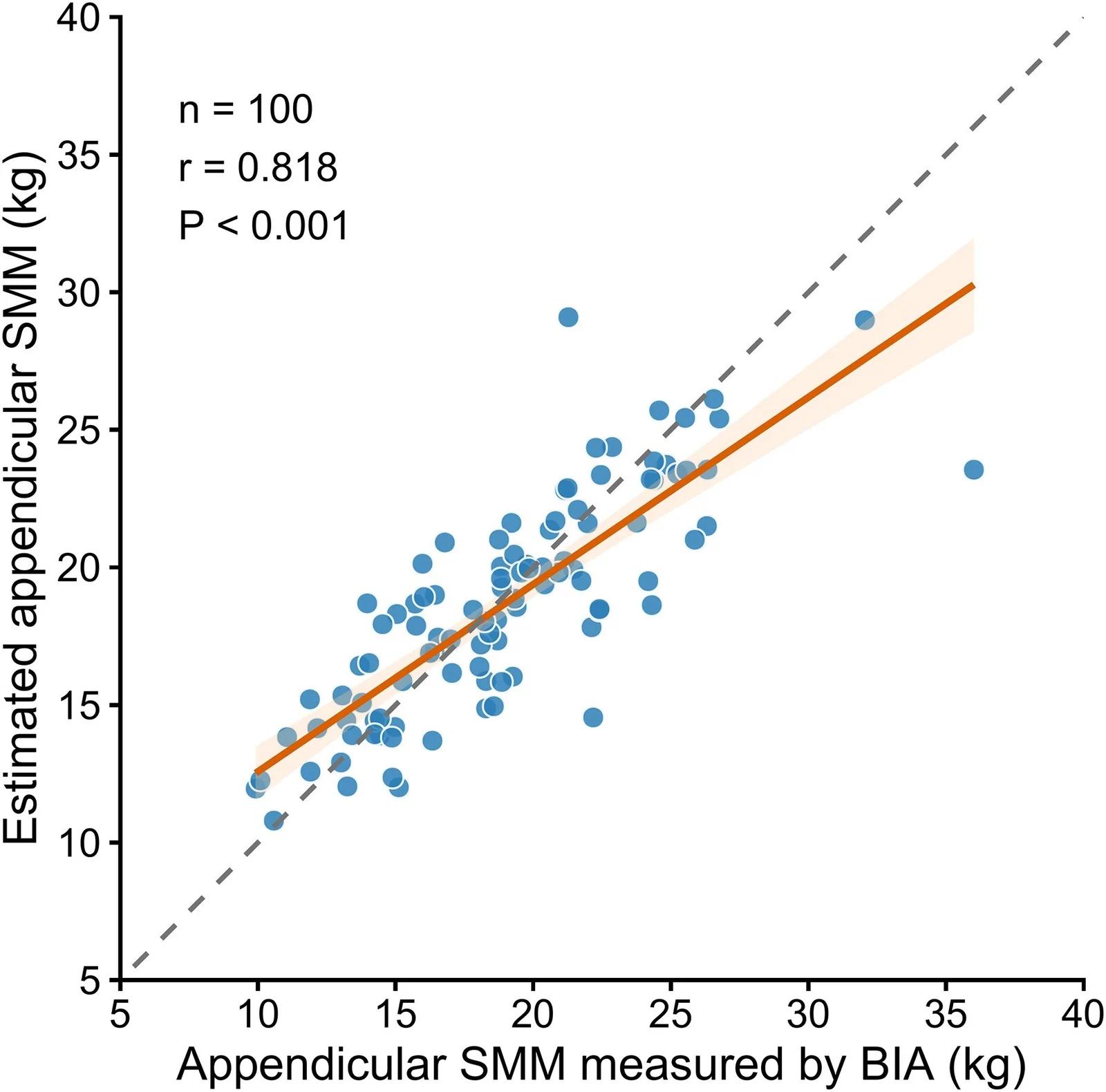
Correlation between appendicular skeletal muscle mass measured by bioelectrical impedance analysis and estimated appendicular skeletal muscle mass by Yamada’s formula. The scatter plot shows the association between appendicular skeletal muscle mass measured by bioelectrical impedance analysis and estimated appendicular skeletal muscle mass calculated using the newly derived equation in the derivation cohort (*n* = 100). The solid line indicates the linear regression line, and the shaded area indicates the 95% confidence interval. The dashed line indicates the line of identity. Pearson’s correlation coefficient was 0.818 (*P* < .001). Abbreviation: SMM, skeletal muscle mass.

### Characteristics of the patients examined (FKR study)

A total of 1403 patients from the FKR study were analyzed. Their characteristics are described in Supplementary Table S1. Briefly, the median age was 70.0 years and 56.3% were men. The proportion of patients with diabetic nephropathy was 11.5%. The distributions of handgrip strength and estimated SMI are shown in Supplementary Figure S1. The median handgrip strength was 30.7 kg for men and 18.6 kg for women. The median estimated SMI values by Sunayama’s formula were 7.79 kg/m^2^ for men and 6.23 kg/m^2^ for women; corresponding values by Yamada’s formula were 7.56 and 6.22 kg/m^2^.

Table 1 summarizes low handgrip strength, low estimated SMI, and the sarcopenia-related high-risk phenotype stratified by sex. Low handgrip strength was present in 573 patients (40.8%). The numbers of patients with low estimated SMI by the Sunayama and Yamada formulas were 218 (15.5%) and 312 (22.2%), respectively. Hence, the phenotype prevalence was 11.9% and 11.7%, respectively. When stratified by CKD stage, the prevalence increased with advancing CKD stage (*P* for trend <.05; Supplementary Figure S2).

**Table 1:** Number of patients with low handgrip strength, low estimated SMI, and the sarcopenia-related high-risk phenotype.

	Total (*n* = 1403)	Men (*n* = 790)	Women (*n* = 613)	*P*-value
Low handgrip strength, *n* (%)	573 (40.8)	298 (37.7)	275 (44.9)	.007
Low estimated SMI by Sunayama’s formula, *n* (%)	218 (15.5)	100 (12.7)	118 (19.2)	<.001
Low estimated SMI by Yamada’s formula, *n* (%)	312 (22.2)	168 (21.3)	144 (23.5)	.320
Sarcopenia-related high-risk phenotype using Sunayama’s formula, *n* (%)	167 (11.9)	80 (10.1)	87 (14.2)	.020
Sarcopenia-related high-risk phenotype using Yamada’s formula, *n* (%)	164 (11.7)	86 (10.9)	78 (12.7)	.288

Low handgrip strength, low estimated SMI by Sunayama’s formula, and the phenotype defined using Sunayama’s formula were more prevalent in women than in men; no significant sex differences were observed for the corresponding Yamada-formula classifications. Chi-square tests were used for comparisons between men and women. Low handgrip strength was defined using the Asian Working Group for Sarcopenia 2019 cutoffs. Low estimated SMI was defined using the AWGS 2019 muscle mass cutoffs after estimating SMI with either Sunayama’s formula or Yamada’s formula. The sarcopenia-related high-risk phenotype was defined as the combination of low handgrip strength and low estimated SMI. *P*-values are shown for descriptive comparisons; no formal correction for multiple comparisons was applied. Abbreviations: AWGS, Asian Working Group for Sarcopenia; SMI, skeletal muscle index.

The characteristics of patients with and without the phenotype are shown in Table 2. Those with the phenotype were significantly older, had a significantly higher prevalence of cardiovascular disease, and had significantly lower body weight, blood hemoglobin, serum albumin, low-density lipoprotein cholesterol, and eGFR. Serum creatinine, serum cystatin C, and phosphorus were significantly higher in those with the phenotype. These findings indicate that the phenotype captured a clinically vulnerable group.

**Table 2:** Baseline characteristics stratified by the presence or absence of the sarcopenia-related high-risk phenotype.

	Phenotype defined by Sunayama’s formula			Phenotype defined by Yamada’s formula		
Baseline data	Phenotype (+)	Phenotype (−)	*P*-value	Phenotype (+)	Phenotype (−)	*P*-value
Patient number	167	1236		164	1239	
Demographic data						
Age, years	81.0 [74.0, 85.0]	69.0 [60.0, 77.0]	<.001	78.5 [70.8, 84.0]	69.0 [60.0, 77.0]	<.001
Sex (male), %	47.9	57.4	.025	52.4	56.8	.315
Diabetes mellitus, %	28.1	29.5	.786	31.1	29.1	.648
CVD history, %	37.7	25.8	.002	36.6	26	.005
History of bone fracture, %	15	5.6	<.001	11.6	6.1	.012
Height, cm	150.0 [146.3, 158.0]	160.0 [154.0, 167.0]	<.001	158.0 [150.0, 165.0]	160.0 [153.0, 166.6]	.059
Body weight, kg	44.9 [40.5, 50.9]	61.4 [54.2, 69.3]	<.001	48.4 [41.1, 54.4]	61.0 [53.6, 69.3]	<.001
Systolic blood pressure, mmHg	131 [119, 144]	132 [121, 144]	.315	130 [116, 144]	132 [121, 144]	.046
Blood tests						
Urinary protein-to-urinary Cr ratio, g/gCr	0.39 [0.16, 1.13]	0.34 [0.10, 1.22]	.245	0.44 [0.16, 1.34]	0.34 [0.10, 1.18]	.039
Hemoglobin, g/dl	11.5 [10.5, 12.6]	12.6 [11.3, 14.0]	<.001	11.3 [10.4, 12.1]	12.7 [11.3, 14.0]	<.001
Serum albumin, g/dl	4.0 [3.7, 4.3]	4.1 [3.9, 4.4]	<.001	4.0 [3.7, 4.3]	4.1 [3.9, 4.4]	.005
Serum hsCRP, mg/dl	0.05 [0.02, 0.13]	0.06 [0.03, 0.13]	.044	0.06 [0.02, 0.13]	0.06 [0.03, 0.13]	.254
Serum HDL-cholesterol, mg/dl	60 [50, 73]	55 [44, 68]	<.001	60 [49, 73]	55 [44, 68]	.003
Serum LDL-cholesterol, mg/dl	101 [82, 123]	109 [88, 130]	.009	101 [79, 123]	109 [89, 130]	.003
Serum Cr, mg/dl	1.45 [1.06, 2.16]	1.31 [0.92, 1.99]	.012	1.56 [1.11, 2.26]	1.30 [0.91, 1.98]	<.001
eGFR, ml/min/1.73 m^2^	30.4 [20.3, 43.1]	39.1 [24.2, 55.8]	<.001	28.9 [18.5, 42.9]	39.5 [24.2, 56.0]	<.001
Serum CystC, mg/l	1.98 [1.48, 2.70]	1.50 [1.01, 2.23]	<.001	2.18 [1.53, 2.90]	1.49 [1.01, 2.21]	<.001
Serum calcium, mg/dl	9.3 [8.9, 9.6]	9.4 [9.1, 9.7]	.069	9.2 [8.9, 9.6]	9.4 [9.1, 9.7]	.009
Serum phosphorus, mg/dl	3.6 [3.2, 4.0]	3.4 [3.0, 3.8]	.003	3.6 [3.2, 4.0]	3.4 [3.0, 3.9]	.001
Plasma intact PTH, pg/ml	78 [55, 123]	68 [47, 110]	.032	84 [51, 124]	67 [47, 110]	.033
Medications						
RAAS inhibitors, %	65.9	70.2	.282	68.9	69.8	.857
Anti-platelet agents, %	31.1	20.9	.004	29.9	21.1	.012
Statin, %	40.7	38.8	.673	39	39	.999

Data are expressed as median [interquartile range] or percentage, as appropriate. The phenotype indicates the sarcopenia-related high-risk phenotype defined as low handgrip strength plus low estimated SMI. *P*-values are shown for descriptive comparisons; no formal correction for multiple comparisons was applied. Abbreviations: Cr, creatinine; CVD, cardiovascular disease; CystC, cystatin C; eGFR, estimated glomerular filtration rate; HDL, high-density lipoprotein; hsCRP, high-sensitivity C-reactive protein; LDL, low-density lipoprotein; PTH, parathyroid hormone; RAAS, renin-angiotensin-aldosterone system; SMI, skeletal muscle index.

### Association between the sarcopenia-related phenotype and outcomes

The median follow-up period was 5 years. One hundred seventy-three patients died during this period, including 43 cardiovascular deaths. The 1-, 3-, and 5-year mortality rates were 1.5%, 8.6%, and 13.8%, respectively. Kaplan-Meier analysis showed significant differences in all-cause mortality between patients with and without the phenotype defined by either formula (Supplementary Figures S3A and S3B). Unadjusted and age- and sex-adjusted Cox models showed that the phenotype was significantly associated with all-cause mortality using each formula (Table 3). This relationship remained significant after adjustment for multiple confounders (Sunayama’s formula: adjusted HR, 1.54; 95% CI, 1.07–2.21; Yamada’s formula: adjusted HR, 2.14; 95% CI, 1.52–3.00; Table 3). The phenotype was associated with cardiovascular mortality using both formulas; for noncardiovascular mortality, the association remained significant only with Yamada’s formula (Table 3).

**Table 3:** HRs (95% CIs) for mortality in patients with the sarcopenia-related high-risk phenotype compared with patients without the phenotype (*n* = 1403).

	HR (95% CI)		HR (95% CI)		HR (95% CI)	
Phenotype defined by Sunayama’s formula	Unadjusted model	*P*-value	Age- and sex-adjusted model	*P*-value	Multivariable-adjusted model	*P*-value
All-cause mortality	4.03 (2.93–5.56)	<.001	1.56 (1.10–2.22)	.013	1.54 (1.07–2.21)	0.020
Cardiovascular mortality	4.96 (2.68–9.17)	<.001	2.09 (1.09–4.00)	.023	2.21 (1.09–4.46)	0.028
Noncardiovascular mortality	3.71 (2.55–5.40)	<.001	1.63 (1.13–2.34)	.008	1.36 (0.89–2.07)	0.158
	HR (95% CI)		HR (95% CI)		HR (95% CI)	
Phenotype defined by Yamada’s formula	Unadjusted model	*P*-value	Age- and sex-adjusted model	*P*-value	Multivariable-adjusted model	*P*-value
All-cause mortality	4.40 (3.20–6.07)	<.001	2.30 (1.65–3.21)	<.001	2.14 (1.52–3.00)	<0.001
Cardiovascular mortality	4.00 (2.09–7.65)	<.001	2.03 (1.06–3.88)	.032	1.99 (1.00–3.94)	0.049
Noncardiovascular mortality	4.50 (3.11–6.51)	<.001	1.62 (1.13–2.33)	.009	2.16 (1.46–3.20)	<0.001

Hazard ratios and 95% confidence intervals for mortality were estimated using unadjusted, age- and sex-adjusted, and multivariable-adjusted Cox proportional hazards regression analyses. The sarcopenia-related high-risk phenotype was defined as low handgrip strength plus low estimated SMI. The following covariates were included in the multivariable-adjusted models: age, sex, presence of diabetic nephropathy, history of cardiovascular disease, systolic blood pressure, eGFR, log-transformed urinary protein-to-creatinine ratio, hemoglobin, albumin, and log-transformed high-sensitivity C-reactive protein.

A two-tailed *P*-value <.05 was considered statistically significant.

Abbreviations: CI, confidence interval; eGFR, estimated glomerular filtration rate; HR, hazard ratio; hsCRP, high-sensitivity C-reactive protein; SMI, skeletal muscle index.

### Secondary and sensitivity analyses

To determine whether handgrip strength and estimated SMI were independently associated with outcomes, multivariable analyses were conducted using these variables as categorical or continuous variables. Lower handgrip strength or lower estimated SMI was generally associated with increased all-cause mortality (Supplementary Tables S2 and S3; Supplementary Figure S4). In four-category analyses, patients with combined low handgrip strength and low estimated SMI had the highest mortality risk, whereas dynapenia also conferred elevated risk (Fig. 2 and Supplementary Figure S5). In the multivariable-adjusted model, the combined group showed HRs of 2.15 (95% CI, 1.34–3.44) using Sunayama’s formula and 3.25 (95% CI, 1.99–5.30) using Yamada’s formula. Adding the phenotype to a base clinical model modestly improved prognostic performance, particularly for Yamada’s formula (Supplementary Table S4). The improvement was modest, supporting use of the phenotype as an adjunct to, rather than a replacement for, conventional clinical risk assessment. Subgroup associations were generally consistent across baseline characteristics; a significant interaction with proteinuria was observed only for Yamada’s formula (*P* for interaction = .011; Supplementary Table S5). Sensitivity analyses using AWGS 2025 age-specific cutoffs and excluding CKD stage G5 yielded consistent associations (Supplementary Tables S6 and S7).

**Figure 2:**
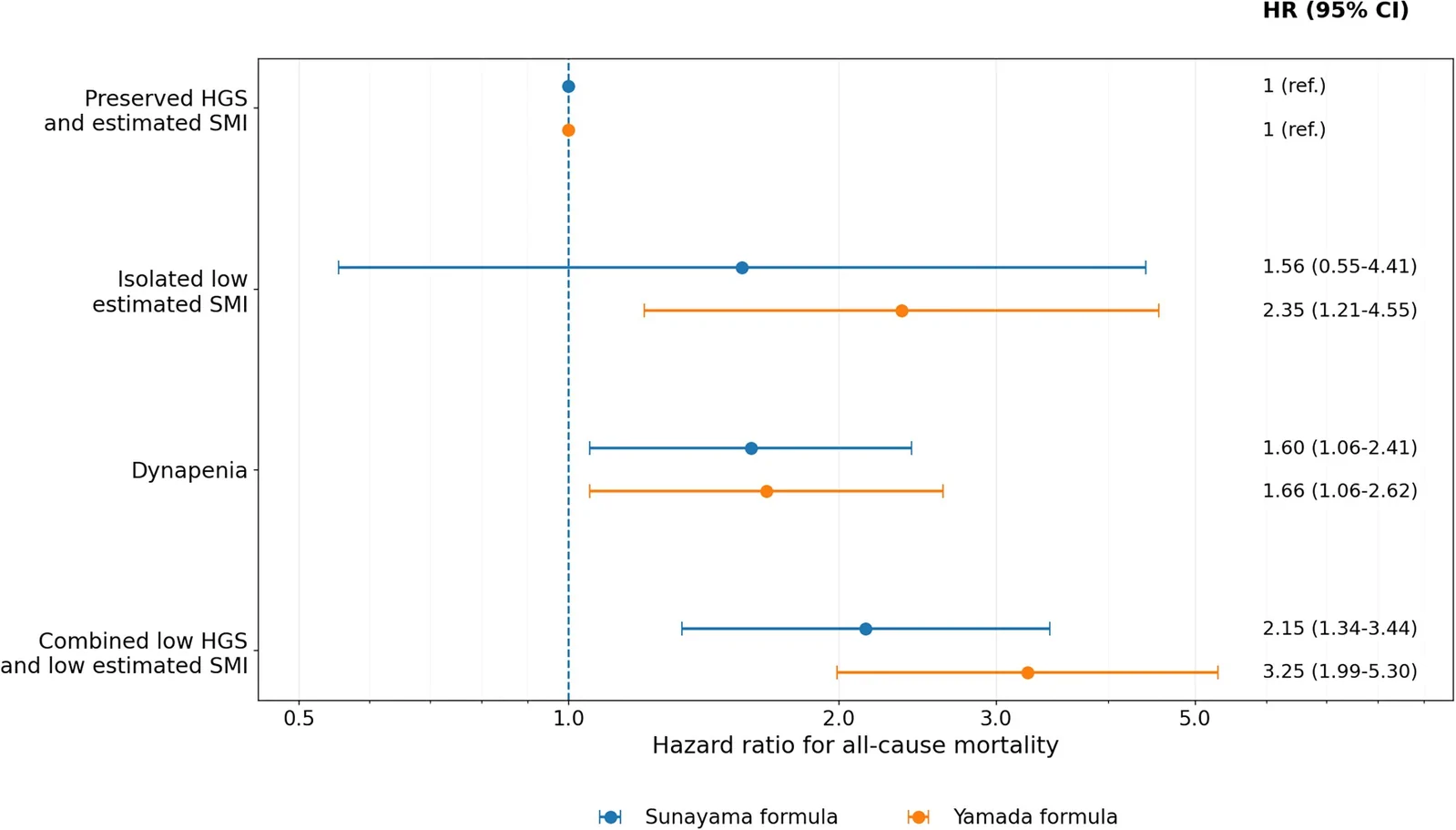
Multivariable-adjusted hazard ratios for all-cause mortality according to the combination of handgrip strength and estimated SMI. Participants were classified into four groups according to HGS and estimated SMI: preserved handgrip strength and estimated SMI, isolated low estimated SMI, dynapenia, and combined low handgrip strength and low estimated SMI. Hazard ratios and 95% confidence intervals were estimated using multivariable-adjusted Cox proportional hazards regression models. Preserved handgrip strength and estimated SMI were used as the reference group. The multivariable model included age, sex, presence of diabetic nephropathy, history of cardiovascular disease, systolic blood pressure, estimated glomerular filtration rate, log-transformed urinary protein-to-creatinine ratio, hemoglobin level, serum albumin level, and log-transformed high-sensitivity C-reactive protein. Abbreviations: CI, confidence interval; HGS, handgrip strength; HR, hazard ratio; SMI, skeletal muscle index.

## DISCUSSION

This study presents a practical approach to identifying a sarcopenia-related high-risk phenotype by integrating a blood test-based estimation of SMI with handgrip strength. We demonstrated that the phenotype, defined as low handgrip strength combined with low estimated SMI, was independently associated with all-cause and cardiovascular mortality in non–dialysis-dependent CKD patients. The four-category analysis further showed that patients with both low handgrip strength and low estimated SMI had the highest mortality risk, whereas dynapenia alone was also associated with higher mortality. Overall, our findings indicate that serum creatinine-serum cystatin C-based surrogates may help stratify muscle-related mortality risk when dual-energy X-ray absorptiometry or bioelectrical impedance analysis is unavailable. However, because direct muscle mass measurement and physical performance were unavailable in the FKR cohort, this approach should not be interpreted as a replacement for guideline-based sarcopenia diagnosis.

Recent work indicates that serum creatinine and serum cystatin C capture complementary biological signals: serum creatinine reflects both kidney function and skeletal muscle mass, whereas serum cystatin C tracks kidney function with less influence from muscle mass [28]. Their combination, particularly as a ratio, may help attenuate confounding by kidney function and isolate a muscle-related signal. In our study, the equations yielded estimated SMI values that, when paired with handgrip strength thresholds, identified a phenotype associated with mortality. Nevertheless, these biomarkers are also influenced by kidney function, inflammation, nutrition, and systemic illness. Thus, estimated SMI may reflect broader disease severity in addition to muscle mass. This limitation motivated the sensitivity analysis excluding CKD stage G5 and the incremental value analysis beyond conventional clinical markers.

In the present cohort, patients with the phenotype had elevated risks of all-cause and cardiovascular mortality. They were also older and had lower body weight, serum albumin, hemoglobin, and eGFR, suggesting consistency with frailty and protein-energy wasting [29]. The mechanisms linking muscle-related vulnerability to mortality in CKD are likely multifactorial. Sedentary behavior, protein-energy wasting, chronic inflammation, and loss of myokine-mediated effects may contribute through cardiovascular events, infection, fractures, and frailty-related complications [30–34]. More broadly, skeletal muscle is increasingly recognized as an organ relevant to health and disease, and muscle mass, strength, and physical performance are linked to activities of daily living [35, 36].

Recent studies have shown that loss of strength does not always parallel a reduction in skeletal muscle mass and is independently associated with adverse outcomes [6, 8]. In our study, both handgrip strength and estimated SMI were associated with all-cause mortality, indicating that muscle strength and estimated muscle mass provide complementary prognostic information. Consistent with these findings, dynapenia, defined as decreased skeletal muscle strength with preserved estimated SMI [37, 38], was associated with increased mortality. These findings support a four-category framework distinguishing dynapenia, isolated low estimated SMI, and the combined phenotype, rather than relying only on a binary phenotype. This framework also addresses whether handgrip strength and estimated SMI should be considered separately or as a combined construct.

Because assessment of sarcopenia requires skeletal muscle mass or SMI, formulas with high predictive performance are clinically attractive. Multiple serum creatinine/serum cystatin C-based indices or equations have been developed or validated using age, sex, body weight, height, serum creatinine, serum cystatin C, and hemoglobin [13–21, 39]. Sunayama’s formula uses age, body weight, hemoglobin, and the serum creatinine/serum cystatin C ratio, whereas Yamada’s formula uses age, sex, body weight, and the same ratio [16]. Unlike Sunayama’s formula, Yamada’s formula does not require hemoglobin, which may modestly simplify its calculation, although hemoglobin is routinely measured in patients with CKD. Although the association with mortality appeared stronger for Yamada’s formula, this does not establish clinical superiority. Similar phenotype prevalence does not imply identical classification, and external validation, calibration, and head-to-head comparison with other equations and device-based measures are needed before broad implementation.

Another important consideration is that formulas should be tailored to specific ethnicities and nationalities. In the present study, we applied a newly developed formula for Japanese non–dialysis-dependent CKD patients, along with another formula originally designed for Japanese individuals. Because body size and composition are strongly influenced by ethnicity and nationality, the generalizability of these formulas to other populations has not been established. Equations derived from general populations may also yield substantial bias when applied to CKD patients. Future research should validate and refine these formulas in diverse CKD populations.

Our study has several strengths. We leveraged a large prospective multicenter registry of patients with non–dialysis-dependent CKD with excellent follow-up. Baseline assessments were systematic, including handgrip testing and routine laboratory tests. We evaluated two blood test approaches to estimate SMI, added four-category analyses to clarify the contribution of handgrip strength and estimated SMI, and performed incremental value and sensitivity analyses, including AWGS 2025 cutoffs and exclusion of CKD stage G5.

The study’s limitations warrant discussion. Its observational design precludes causal inference. The new equation was derived using bioelectrical impedance analysis as the reference in only 100 patients and was not validated against dual-energy X-ray absorptiometry or computed tomography. Neither equation was revalidated against direct muscle mass assessment in the FKR cohort, preventing assessment of diagnostic accuracy. Decreased physical performance was not considered; thus, misclassification could not be ruled out. Serum creatinine and serum cystatin C are influenced by kidney function and systemic illness, especially in advanced CKD. Furthermore, the generalizability of our findings beyond Japanese patients is uncertain, and residual confounding by physical activity, diet, inflammatory burden, and comorbidity cannot be ruled out. In particular, sensitivity, specificity, and calibration against device-measured muscle mass remain unknown. Future work should incorporate repeated measures, direct comparisons with standard muscle mass assessments, external validation in diverse populations, and intervention-based evaluation.

## CONCLUSION

In non–dialysis-dependent CKD, low handgrip strength combined with low blood test-derived estimated SMI identified a sarcopenia-related high-risk phenotype associated with higher all-cause mortality. This approach may support screening and prognostic risk stratification when direct muscle mass assessment is unavailable in routine nephrology practice, but it should not be considered a diagnostic substitute without further validation.

## Supplementary Material

sfag336_Supplemental_File

## Data Availability

The data underlying this article are not publicly available because of institutional data governance restrictions and the inclusion of potentially identifiable patient information. Data may be made available upon reasonable request to the corresponding author and with approval from the Fukuoka Kidney disease Registry (FKR) Data Management Committee.
