# Supplementary material for: A blood test-derived skeletal muscle index combined with handgrip strength identifies a sarcopenia-related high-risk phenotype in non–dialysis-dependent chronic kidney disease
Source: Clin Kidney J. 2026 Sep 25;19(10):sfag336. doi: 10.1093/ckj/sfag336 (PMC13655739; doi:10.1093/ckj/sfag336)
Supplement: Supplementary file 1 [file sfag336_supplemental_file.docx]

**SUPPLEMENTARY MATERIALS**

**A blood test-derived skeletal muscle index combined with handgrip strength identifies a sarcopenia-related high-risk phenotype in non-dialysis-dependent chronic kidney disease**

**Authors**

Shunsuke Yamada, M.D., Ph.D.,^1,2^ Shigeru Tanaka, M.D., Ph.D.,^1^ Kenji Harada, M.D.,^3^ Hisako Yoshida, Ph.D.,^4^ Hiromasa Kitamura, M.D., Ph.D.,^1^ Hokuto Arase, M.D., Ph.D.,^5^ Masumi Shojima, M.D.,^1^ Kazuhiko Tsuruya, M.D., Ph.D.,^6^ Toshiaki Nakano, M.D., Ph.D.,^1,2^ and Tetsuro Ago, M.D., Ph.D.^1^,^7^

**Affiliations**

^1^Department of Medicine and Clinical Science, Graduate School of Medical Sciences, Kyushu University, Fukuoka, Japan

^2^ Division of Kidney Care Unit, Kyushu University Hospital, Fukuoka, Japan

^3^ Department of Nephrology, Kokura Memorial Hospital, Kitakyushu, Japan

^4^ Department of Medical Statistics, Osaka Metropolitan University Graduate School of Medicine, Osaka, Japan

^5^ Division of Nephrology, National Hospital Organization Fukuoka-Higashi Medical Center, Koga, Japan

^6^ Department of Nephrology, Nara Medical University, Kashihara, Japan

^7^ Center for Cohort Studies, Graduate School of Medical Sciences, Kyushu University, Fukuoka, Japan

**Corresponding Author:**

Shunsuke Yamada, M.D., Ph.D.,

Department of Medicine and Clinical Science, Graduate School of Medical Sciences, Kyushu University, 3-1-1 Maidashi, Higashi-Ku, Fukuoka 8128582, Japan.

Phone: +81-92-642-5843; Fax: +81-92-642-5846

E-mail: [shunsuke.yamada.944@m.kyushu-u.ac.jp](mailto:shunsuke.yamada.944@m.kyushu-u.ac.jp)

**SUPPLEMENTARY TABLES**

**Supplementary Table S1. Baseline characteristics (n=1,403).**

| **Characteristic** | **Value** |
| --- | --- |
| **Demographic data** |  |
| Age, years | 70.0 (62.0-78.0) |
| Sex (male), % | 56.3 |
| CVD history, % | 27.2 |
| Diabetic nephropathy, % | 11.5 |
| Height, cm | 160 (153-167) |
| Body weight, kg | 59.4 (51.6-68.1) |
| Body mass index, kg/m² | 23.3 (20.9-26.1) |
| Systolic blood pressure, mmHg | 132 (120-144) |
| **Blood and urine tests** |  |
| Urinary protein to creatinine excretion, g/gCr | 0.35 (0.11-1.21) |
| Blood hemoglobin, g/dL | 12.5 (11.2-13.9) |
| Serum albumin, g/dL | 4.1 (3.9-4.4) |
| Serum hsCRP, mg/dL | 0.06 (0.03-0.13) |
| Serum creatinine, mg/dL | 1.33 (0.94-2.02) |
| eGFR, mL/min/1.73 m² | 37.8 (23.6-54.6) |
| Serum cystatin C, mg/L | 1.55 (1.04-2.31) |
| Serum calcium, mg/dL | 9.4 (9.1-9.7) |
| Serum phosphorus, mg/dL | 3.4 (3.0-3.9) |
| Plasma intact PTH, pg/mL | 69 (47-114) |
| **Medications** |  |
| RAAS inhibitors, % | 69.7 |
| Anti-platelet agents, % | 22.1 |
| Statin, % | 39.0 |

Data are expressed as median (interquartile range) or percentage as appropriate. Abbreviations: Cr, creatinine; CVD, cardiovascular disease; eGFR, estimated glomerular filtration rate; hsCRP, high-sensitivity C-reactive protein; PTH, parathyroid hormone; RAAS, renin-angiotensin-aldosterone system.

**Supplementary Table S2. Multivariable-adjusted HRs (95% CIs) for all-cause mortality stratified by quartiles of handgrip strength and estimated SMI calculated using two formulas.**

| **Marker** | **Quartile** | **Event No/Patient No** | **HR (95% CI)** | ***P*-value** | ***P* for trend** |
| --- | --- | --- | --- | --- | --- |
| **Handgrip strength** |  |  |  |  |  |
|  | Q1 | 72/348 | 2.35 (1.20-4.60) | 0.013 |  |
|  | Q2 | 45/352 | 1.54 (0.82-2.88) | 0.181 | 0.003 |
|  | Q3 | 40/352 | 1.19 (0.65-2.17) | 0.573 |  |
|  | Q4 | 16/351 | 1 (ref.) |  |  |
| **Estimated SMI (Sunayama formula)** |  |  |  |  |  |
|  | Q1 | 54/350 | 1.83 (0.95-3.49) | 0.069 |  |
|  | Q2 | 49/351 | 1.07 (0.64-1.80) | 0.800 | 0.108 |
|  | Q3 | 42/350 | 0.99 (0.61-1.63) | 0.999 |  |
|  | Q4 | 28/352 | 1 (ref.) |  |  |
| **Estimated SMI (Yamada formula)** |  |  |  |  |  |
|  | Q1 | 48/350 | 2.04 (1.18-3.54) | 0.011 |  |
|  | Q2 | 53/352 | 1.29 (0.80-2.06) | 0.294 | 0.009 |
|  | Q3 | 39/350 | 0.93 (0.58-1.50) | 0.770 |  |
|  | Q4 | 33/351 | 1 (ref.) |  |  |

Patients were divided into quartiles according to baseline values of handgrip strength or estimated SMI calculated using two formulas. HRs and 95% CIs for all-cause mortality were estimated using multivariable-adjusted Cox proportional hazards regression models. Covariates included age, sex, presence of diabetic nephropathy, history of cardiovascular disease, systolic blood pressure, eGFR, log-transformed urinary protein-to-creatinine ratio, hemoglobin, albumin, and log-transformed hsCRP. Abbreviations: CI, confidence interval; eGFR, estimated glomerular filtration rate; HR, hazard ratio; hsCRP, high-sensitivity C-reactive protein; SMI, skeletal muscle index.

**Supplementary Table S3. Multivariable-adjusted HRs (95% CIs) for all-cause mortality stratified by sex-specific quartiles of handgrip strength and estimated SMI calculated using two formulas.**

| **Marker** | **Sex-specific quartile** | **Event No/Patient No** | **HR (95% CI)** | ***P*-value** | ***P* for trend** |
| --- | --- | --- | --- | --- | --- |
| **Handgrip strength** |  |  |  |  |  |
|  | Q1 | 104/350 | 1.44 (0.77-2.71) | 0.253 |  |
|  | Q2 | 39/348 | 0.81 (0.43-1.54) | 0.518 | 0.001 |
|  | Q3 | 16/355 | 0.52 (0.25-1.10) | 0.086 |  |
|  | Q4 | 14/350 | 1 (ref.) |  |  |
| **Estimated SMI (Sunayama formula)** |  |  |  |  |  |
|  | Q1 | 88/350 | 2.26 (1.27-4.00) | 0.005 |  |
|  | Q2 | 39/350 | 1.56 (0.85-2.85) | 0.150 | 0.001 |
|  | Q3 | 30/351 | 1.39 (0.75-2.58) | 0.291 |  |
|  | Q4 | 16/352 | 1 (ref.) |  |  |
| **Estimated SMI (Yamada formula)** |  |  |  |  |  |
|  | Q1 | 73/350 | 1.69 (1.08-2.63) | 0.021 |  |
|  | Q2 | 33/351 | 0.75 (0.45-1.24) | 0.259 | 0.010 |
|  | Q3 | 35/351 | 0.91 (0.56-1.48) | 0.705 |  |
|  | Q4 | 32/351 | 1 (ref.) |  |  |

Patients were divided into sex-specific quartiles according to baseline values of handgrip strength or estimated SMI calculated using two formulas. HRs and 95% CIs for all-cause mortality were estimated using multivariable-adjusted Cox proportional hazards regression models. Covariates included age, sex, presence of diabetic nephropathy, history of cardiovascular disease, systolic blood pressure, eGFR, log-transformed urinary protein-to-creatinine ratio, hemoglobin, albumin, and log-transformed hsCRP. Abbreviations: CI, confidence interval; eGFR, estimated glomerular filtration rate; HR, hazard ratio; hsCRP, high-sensitivity C-reactive protein; SMI, skeletal muscle index.

**Supplementary Table S4. Incremental prognostic value of the sarcopenia-related phenotype beyond conventional clinical markers.**

| **Formula** | **N** | **Events** | **C-index,**  **base model** | **C-index, extended model** | **Δ C-index** | **AIC,**  **base model** | **AIC,**  **extended model** | **Δ AIC** | **LRT *P*-value** |
| --- | --- | --- | --- | --- | --- | --- | --- | --- | --- |
| Sunayama formula | 1,403 | 173 | 0.850 | 0.853 | 0.003 | 2137.54 | 2134.31 | -3.24 | 0.022 |
| Yamada formula | 1,403 | 173 | 0.850 | 0.857 | 0.007 | 2137.54 | 2121.91 | -15.64 | <0.001 |

The base model included age, sex, presence of diabetic nephropathy, history of cardiovascular disease, systolic blood pressure, eGFR, log-transformed urinary protein-to-creatinine ratio, hemoglobin, albumin, and log-transformed high-sensitivity C-reactive protein. AIC, Akaike information criterion; LRT, likelihood ratio test.

**Supplementary Table S5. Subgroup analysis for the association between the sarcopenia-related phenotype and all-cause mortality across baseline characteristics.**

| **Formula/Subgroup** | **Category** | **Event No/Total No** | **HR (95% CI)** | ***P*-value** | ***P* for interaction** |
| --- | --- | --- | --- | --- | --- |
| **Sunayama formula** |  |  |  |  |  |
| Age | <70 years | 17/660 | 5.98 (1.20-29.81) | 0.029 | 0.192 |
|  | ≥70 years | 156/743 | 1.54 (1.06-2.22) | 0.024 |  |
| Sex | Female | 58/613 | 2.21 (1.24-3.95) | 0.007 | 0.067 |
|  | Male | 115/790 | 1.21 (0.75-1.96) | 0.426 |  |
| Diabetes mellitus | Absence | 114/991 | 1.46 (0.93-2.29) | 0.101 | 0.890 |
|  | Presence | 59/412 | 1.77 (0.93-3.38) | 0.083 |  |
| Proteinuria | <1.0 g/gCr | 116/986 | 1.96 (1.26-3.05) | 0.003 | 0.109 |
|  | ≥1.0 g/gCr | 57/417 | 0.87 (0.45-1.69) | 0.679 |  |
| eGFR | <30 mL/min/1.73 m² | 104/529 | 1.32 (0.82-2.13) | 0.251 | 0.255 |
|  | ≥30 mL/min/1.73 m² | 69/874 | 1.99 (1.09-3.62) | 0.024 |  |
| **Yamada formula** |  |  |  |  |  |
| Age | <70 years | 17/660 | 2.73 (0.70-10.70) | 0.149 | 0.791 |
|  | ≥70 years | 156/743 | 2.12 (1.49-3.02) | <0.001 |  |
| Sex | Female | 58/613 | 3.08 (1.73-5.50) | <0.001 | 0.053 |
|  | Male | 115/790 | 1.69 (1.09-2.62) | 0.019 |  |
| Diabetes mellitus | Absence | 114/991 | 2.52 (1.67-3.83) | <0.001 | 0.123 |
|  | Presence | 59/412 | 1.73 (0.91-3.28) | 0.094 |  |
| Proteinuria | <1.0 g/gCr | 116/986 | 2.90 (1.94-4.35) | <0.001 | 0.011 |
|  | ≥1.0 g/gCr | 57/417 | 1.01 (0.50-2.04) | 0.974 |  |
| eGFR | <30 mL/min/1.73 m² | 104/529 | 2.02 (1.30-3.12) | 0.002 | 0.334 |
|  | ≥30 mL/min/1.73 m² | 69/874 | 2.41 (1.35-4.32) | 0.003 |  |

HRs and 95% CIs for all-cause mortality were estimated using multivariable-adjusted Cox proportional hazards regression models. Covariates included age, sex, presence of diabetic nephropathy, history of cardiovascular disease, systolic blood pressure, log-transformed urinary protein-to-creatinine ratio, hemoglobin, eGFR, albumin, and log-transformed hsCRP. Abbreviations: CI, confidence interval; Cr, creatinine; eGFR, estimated glomerular filtration rate; HR, hazard ratio; hsCRP, high-sensitivity C-reactive protein

**Supplementary Table S6. Sensitivity analysis using AWGS 2025 age-specific cutoffs.**

| **Formula** | **N** | **Events** | **Low HGS,**  **n (%)** | **Low estimated SMI,**  **n (%)** | **Sarcopenia-related phenotype, n (%)** | **Deaths in phenotype, n (%)** | **Multivariable-adjusted HR (95% CI)** | ***P*-value** |
| --- | --- | --- | --- | --- | --- | --- | --- | --- |
| Sunayama formula | 1,217 | 172 | 598 (49.1) | 232 (19.1) | 182 (15.0) | 55 (30.2) | 1.50 (1.05-2.15) | 0.026 |
| Yamada formula | 1,217 | 172 | 598 (49.1) | 312 (25.6) | 180 (14.8) | 55 (30.6) | 2.04 (1.46-2.86) | <0.001 |

This post-hoc sensitivity analysis was restricted to participants aged 50 years or older. AWGS 2025 age-specific cutoffs were applied to define low handgrip strength and low estimated SMI. The multivariable model included the same covariates as the primary analysis. AWGS, Asian Working Group for Sarcopenia; CI, confidence interval; HGS, handgrip strength; HR, hazard ratio; SMI, skeletal muscle index.

**Supplementary Table S7. Sensitivity analysis excluding patients with CKD stage G5.**

| **Formula** | **N** | **Events** | **Sarcopenia-related phenotype, n (%)** | **Deaths in phenotype, n (%)** | **Deaths without phenotype, n (%)** | **Multivariable-adjusted**  **HR (95% CI)** | ***P*-value** |
| --- | --- | --- | --- | --- | --- | --- | --- |
| Sunayama formula | 1,261 | 150 | 147 (11.7) | 48 (32.7) | 102 (9.2) | 1.51 (1.02-2.24) | 0.039 |
| Yamada formula | 1,261 | 150 | 138 (10.9) | 47 (34.1) | 103 (9.2) | 2.13 (1.47-3.07) | <0.001 |

Patients with eGFR <15 mL/min/1.73 m² were excluded. The multivariable model included the same covariates as the primary analysis. CI, confidence interval; CKD, chronic kidney disease; eGFR, estimated glomerular filtration rate; HR, hazard ratio.

**SUPPLEMENTARY FIGURES**

**Supplementary Figure S1.** Distributions of handgrip strength and estimated SMI stratified by sex.

Distributions are shown for (A) handgrip strength, (B) estimated SMI calculated using Sunayama’s formula, and (C) estimated SMI calculated using Yamada’s formula. Vertical dashed lines indicate the sex-specific cutoffs used to define low handgrip strength and low estimated SMI. For handgrip strength, the cutoffs were <28 kg for men and <18 kg for women. For estimated SMI, the cutoffs were <7.0 kg/m^2^ for men and <5.7 kg/m^2^ for women. Abbreviations: HGS, handgrip strength; SMI, skeletal muscle index.

**Supplementary Figure S2.** Prevalence of the sarcopenia-related high-risk phenotype across CKD stages.

The prevalence of the sarcopenia-related high-risk phenotype is shown across CKD stages according to low handgrip strength combined with low estimated SMI calculated using either Sunayama’s formula or Yamada’s formula. The phenotype became more frequent with advancing CKD stage. Abbreviations: CKD, chronic kidney disease; SMI, skeletal muscle index.

**Supplementary Figure S3.** Kaplan–Meier curves for all-cause mortality according to the binary sarcopenia-related high-risk phenotype.

Kaplan–Meier curves are shown for patients with and without the sarcopenia-related high-risk phenotype defined using (A) Sunayama’s formula and (B) Yamada’s formula. The phenotype was defined as combined low handgrip strength and low estimated SMI. Numbers at risk are shown below the x-axis. Differences between groups were assessed using the log-rank test. Abbreviation: SMI, skeletal muscle index.

**Supplementary Figure S4.** Restricted cubic spline curves for handgrip strength and estimated SMI in relation to all-cause mortality.

Restricted cubic spline curves show the multivariable-adjusted hazard ratios and 95% confidence intervals for all-cause mortality according to (A) handgrip strength, (B) estimated SMI calculated using Sunayama’s formula, and (C) estimated SMI calculated using Yamada’s formula. Four knots were placed at the 5th, 35th, 65th, and 95th percentiles, and the median value was used as the reference. The multivariable model included age, sex, presence of diabetic nephropathy, history of cardiovascular disease, systolic blood pressure, estimated glomerular filtration rate, log-transformed urinary protein-to-creatinine ratio, blood hemoglobin level, serum albumin level, and log-transformed high-sensitivity C-reactive protein. P-values for non-linearity were 0.116 for handgrip strength, 0.012 for estimated SMI by Sunayama’s formula, and 0.006 for estimated SMI by Yamada’s formula. Abbreviations: CI, confidence interval; HR, hazard ratio; SMI, skeletal muscle index.

**Supplementary Figure S5.** Kaplan–Meier curves for all-cause mortality according to the four groups defined by handgrip strength and estimated SMI.

Kaplan–Meier curves are shown for four groups defined using (A) Sunayama’s formula and (B) Yamada’s formula: preserved handgrip strength and estimated SMI, isolated low estimated SMI, dynapenia, and combined low handgrip strength and low estimated SMI. In the classification using Sunayama’s formula, 779 patients had preserved handgrip strength and estimated SMI, 51 had isolated low estimated SMI, 406 had dynapenia, and 167 had combined low handgrip strength and low estimated SMI. In the classification using Yamada’s formula, 682 patients had preserved handgrip strength and estimated SMI, 148 had isolated low estimated SMI, 409 had dynapenia, and 164 had combined low handgrip strength and low estimated SMI. Numbers at risk are shown below the x-axis. Differences among groups were assessed using the log-rank test. Abbreviation: SMI, skeletal muscle index.
